# Observational cohort study to determine the degree and causes of variation in the rate of surgery or primary endocrine therapy in older women with operable breast cancer

**DOI:** 10.1016/j.ejso.2020.09.029

**Published:** 2021-02

**Authors:** Jenna L. Morgan, Geoff Holmes, Sue Ward, Charlene Martin, Maria Burton, Stephen J. Walters, Kwok Leung Cheung, Riccardo A. Audisio, Malcolm WR. Reed, Lynda Wyld, the Bridging the Age Gap Trial Management TeamKaren Collins, Kate Lifford, Adrian Edwards, Kate Brain, Alistair Ring, Thomson Robinson, Tim Chater, Kirsty Pemberton, Anne Shrestha, Anthony Nettleship, Paul Richards, Annaliza Todd, Helena Harder, Juliette Wright, Richard Simcock, Chris Murray, Tracy Green, Deirdre Revill, Jacqui Gath, Kieran Horgan, Chris Holcombe, Jay Naik, Rishi Parmeshwar

**Affiliations:** i Division of Population Medicine, Cardiff University, 8th Floor, Neuadd Meirionnydd, Heath Park, Cardiff, CF14 4YS, UK; j Breast Unit, The Royal Marsden NHS Foundation Trust. Downs Road, Sutton,Surrey, SM2 5PT, UK; k Department of Cardiovascular Sciences, University of Leicester, Cardiovascular Research, UK; l EpiGenesys, University of Sheffield, Floor C, Cathedral Court, 1 Vicar Lane, Sheffield, S1 2LT, UK Centre, Glenfield General Hospital, Leicester, LE3 9QP, UK; m Members of the North Trent Consumer Research Panel (NTCRP), UK; n Leeds General Infirmary, Great George Street, Leeds, West Yorkshire, LS1 3EX, UK; o Liverpool and Broadgreen Hospitals NHS Foundation Trust, Thomas Drive, Liverpool, Merseyside, L14 3LB, UK; p Pinderfields Hospital, Mid Yorkshire NHS Foundation Trust, Aberford Rd, Wakefield, UK; q Royal Lancashire Infirmary Ashton Road, Lancaster, Lancashire, LA1 4RP, UK; r Academic Unit of Surgical Oncology, University of Sheffield Medical School, Beech Hill Road, Sheffield, UK; s Department of Health Economics and Decision Science, School for Health and Related Research, University of Sheffield, Sheffield, UK; t Centre for Health and Social Care Research, Sheffield Hallam University, Collegiate Crescent, Sheffield, UK; u Clinical Trials Research Unit, School for Health and Related Research, ScHARR, University of Sheffield, UK; a Academic Unit of Surgical Oncology, University of Sheffield Medical School, Beech Hill Road, Sheffield, UK; b Department of Health Economics and Decision Science, School for Health and Related Research, University of Sheffield, Sheffield, UK; c Centre for Health and Social Care Research, Sheffield Hallam University, Collegiate Crescent, Sheffield, UK; d Clinical Trials Research Unit, School for Health and Related Research, ScHARR, University of Sheffield, UK; e University of Nottingham, Royal Derby Hospital, Uttoxeter Road, Derby, DE22 3DT, UK; f University of Gothenberg, Sahlgrenska Universitetssjukhuset, 41345, Göteborg, Sweden; g Brighton and Sussex Medical School, Brighton, UK; h Department of Health and Social care, Sheffield Hallam University, Sheffield, UK

**Keywords:** Breast cancer, Elderly, Primary endocrine therapy, Surgery, Treatment variation

## Abstract

**Background:**

In the UK there is variation in the treatment of older women with breast cancer, with up to 40% receiving primary endocrine therapy (PET), which is associated with inferior survival. Case mix and patient choice may explain some variation in practice but clinician preference may also be important.

**Methods:**

A multicentre prospective cohort study of women aged >70 with operable breast cancer. Patient characteristics (health status, age, tumour characteristics, treatment allocation and decision-making preference) were analysed to identify whether treatment variation persisted following case-mix adjustment. Expected case-mix adjusted surgery rates were derived by logistic regression using the variables age, co-morbidity, tumour stage and grade. Concordance between patients’ preferred and actual decision-making style was assessed and associations between age, treatment and decision-making style calculated.

**Results:**

Women (median age 77, range 70–102) were recruited from 56 UK breast units between 2013 and 2018. Of 2854/3369 eligible women with oestrogen receptor positive breast cancer, 2354 were treated with surgery and 500 with PET. Unadjusted surgery rates varied between hospitals, with 23/56 units falling outside the 95% confidence intervals on funnel plots. Adjusting for case mix reduced, but did not eliminate, this variation between hospitals (10/56 units had practice outside the 95% confidence intervals). Patients treated with PET had more patient-centred decisions compared to surgical patients (42.2% vs 28.4%, p < 0.001).

**Conclusions:**

This study demonstrates variation in treatment selection thresholds for older women with breast cancer. Health stratified guidelines on thresholds for PET would help reduce variation, although patient preference should still be respected.

## Introduction

Older women (>70 years) account for more than a third of new breast cancer diagnoses in the UK and have poorer outcomes compared to younger women, with later stage at presentation and higher rates of non-standard treatment [[1], [2], [3], [4]]. One such treatment is Primary Endocrine Therapy (PET), where surgery is omitted in favour of endocrine therapy alone to treat women with oestrogen receptor positive (ER+) breast cancer. A Cochrane review comparing PET with surgery in the over 70s demonstrated superior rates of local control with surgery but no difference in five year overall survival rates [5]. However the included studies were flawed by modern standards as some included women with ER negative disease and some also included younger, healthy women. More recent studies have advocated the use of PET only in the very old or frail [6] and current guidelines state that only patients who decline surgery or who are unfit for surgery should be treated this way [7]. In addition, a more recent individual patient meta-analysis of the randomised trial data, with longer follow-up, conducted by the Early Breast Cancer Trialist's group, demonstrated significantly improved long term (15 years) survival in surgically treated women [8].

Surgery for all older women is not appropriate and may cause harm if offered to the frailest and most comorbid older women. This was recently demonstrated by a study of outcomes of nursing home residents with breast cancer in the USA [9]. These frail older women were all treated with surgery which resulted in significant morbidity and mortality as well as causing significant functional decline. Surgery may also have a negative impact on quality-of-life due to long term adverse events such as lymphoedema and chronic pain. Therefore in frailer older women, for whom life expectancy is limited, PET is potentially the better option. The issue is determining where this threshold sits.

In the UK there is considerable variation in the use of PET to treat women over 70 with operable breast cancer [10], with regional rates varying between 12 and 40% [11,12]. A study using retrospective registry data has shown that case mix (variation in stage, health, fitness, deprivation levels) does not account for all of this variation [13]. This suggests that some of this variation is due to individual surgeon or patient preference. Patient preference for non-surgical therapy is often reported as a major factor in determining PET treatment in older patients [14]. However previous studies examining this have suggested that lower rates of surgery in older patients are unlikely to be due to patient choice alone [15]. Variation due to surgeon preference is substantial [16].

The treatment of older women with operable breast cancer may be considered a preference-sensitive healthcare decision and so it is important that shared decision-making be employed, with patients and healthcare professionals working together to determine the best treatment for that individual based on the clinical evidence and the patients’ informed preferences [17,18]. However, there is some evidence to suggest that not all older patients wish to engage in shared decision-making, preferring instead to simply receive information [19] and accept a doctor-led treatment decision [[20], [21], [22]].

The present study used prospectively collected detailed data from a large, multi-centre cohort study, which examined surgical treatment rates across UK hospitals in older women with operable, ER + breast cancer (before and after adjustment for case-mix). We also investigated associations between treatment choice (surgery or PET) and patient decision-making style to determine whether patient preference was likely to be a significant factor in this variance.

## Methods

### Regulatory approval

Ethics approval and research governance approval was obtained (IRAS: 12 LO 1808). All patients gave written informed consent or consent was given by a proxy if the patient was cognitively impaired. The trial reporting followed the STROBE guidelines for reporting of observational studies [23].

### Study design

A prospective, multicentre, comprehensive observational cohort study.

### Sites

Patients were recruited from 56 breast units in England and Wales (Supplemental Table ST1).

### Inclusion criteria

Female patients ≥70 years of age. Primary operable invasive breast cancer (TNM stages: T1-4, N0-2, M0).

### Exclusion criteria

Disease unsuitable for surgery. Previous breast cancer within five years.

### Baseline data collection

Women were recruited at the time of breast cancer diagnosis and before treatment.

A baseline comprehensive geriatric assessment was performed using a range of validated tools with data collected on age, comorbidities (Charlson comorbidity index; CCI) [24], functional status (activities of daily living; ADL [25] and instrumental activities of daily living; IADL [26]), cognitive function (using the Mini Mental State Examination; MMSE [27]). Cognitive impairment was defined as a MMSE score <24, if they were consented by proxy or if dementia was identified on the CCI. Nutritional status was measured using the abridged patient generated subjective global assessment (aPBSGA) [28].

Baseline tumour characteristics were collected, including tumour size, biological subtype, grade and nodal status (both clinical, imaging and pathological status).

Patients’ preferred and actual decision-making styles for their breast cancer treatment were also recorded using a validated questionnaire instrument [29,30]. The questionnaire instrument uses a five point scale for both preferred and actual decision-making styles, ranging from the doctor making all decisions, through to the patient making the final decisions (see Supplemental Table ST2). The decision-making preferences questionnaire was applied within 4 weeks of diagnosis and prior to treatment. Decision-making styles were then classified into one of three categories: Patient-centred, Shared and Doctor-centred (see Supplemental Table ST2).

### Statistical analyses

Primary treatment was dichotomised as surgery or PET. The proportion of patients undergoing surgery was calculated for each hospital.

Multivariable logistic regression was used to estimate the probability of a woman undergoing surgical treatment based on patient level factors, including age, Charlson co-morbidity index, activities of daily living (ADL), instrumental activities of daily living (IADL), Eastern Cooperative Oncology Group (ECOG) performance status, tumour size and grade. Univariate models were first built including all variables and the model AIC values were used to determine which variables had most predictive importance. Multivariate models were then formed by adding variables in order of importance until the model AIC value ceased to improve. Further tests adding and removing individual covariates and comparing AIC led to a preferred model, which explained but did not over-fit the data. Missing data on disease characteristics and co-morbidity was handled using the method of multiple imputation by chained equations (MICE) [31] to produce 25 imputed data sets and combining the results [32].

Expected rates of surgical treatment were calculated for each hospital by summing the individual patient probabilities estimated from the logistic regression model. Risk adjusted rates of surgery were produced by dividing the observed rate by the expected rate for each clinician and hospital and multiplying this by the national rate [33].

Both unadjusted and adjusted rates of surgery at hospital level were displayed graphically as funnel plots to allow examination of the variability at each level and identification of outlying practice. Funnel plots contain two limits; under the hypothesis that treatment choice is randomly determined and independent of clinician or hospital, 95% of units would be expected to lie within the inner limits (2 standard deviations from the mean) and 99% within the outer limits (3 standard deviations from the mean). Hospitals were said to have a Low Surgery rate if they lay below the 95% CI after adjustment for case mix and High Surgery rate if they lay above the 95% CI after adjustment for case mix.

Concordance between preferred and actual decision-making preferences was assessed using Kappa and association between treatment, patient characteristics and decision-making style were identified using Chi-squared tests. Statistical significance was taken at p < 0.05.

Logistic regressions and multiple imputations were performed using the open source statistical programming language R (version 3.0.1), with the remaining data handling and analysis performed in Microsoft Excel for Windows 10.

## Results

### Baseline characteristics

**Fig. 1:**
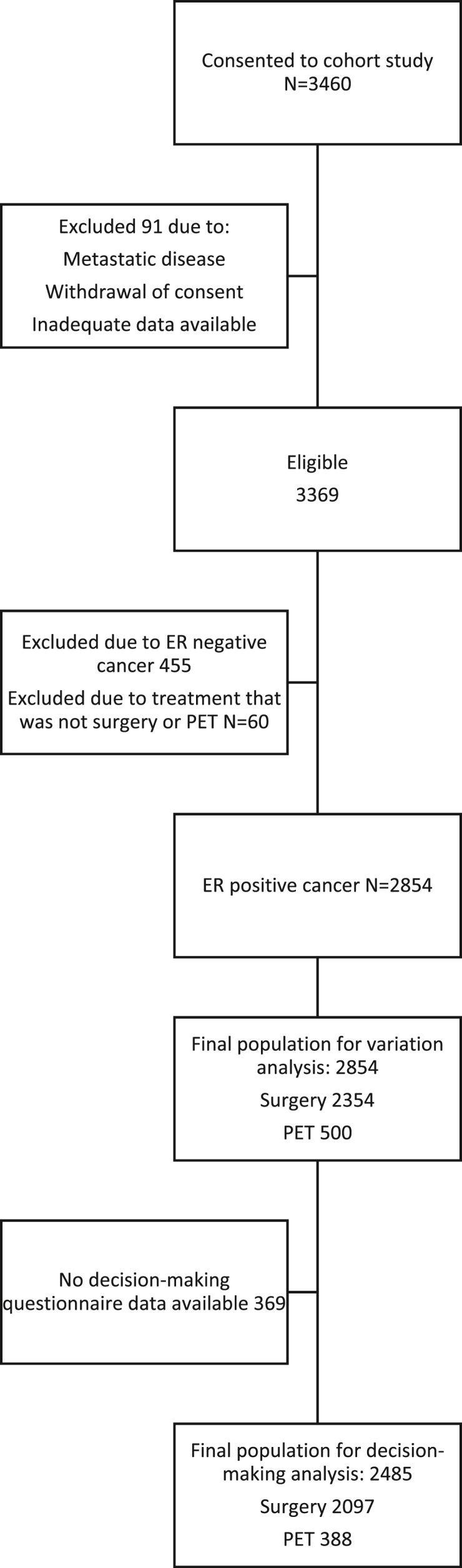
Flow diagram for study.

A total of 3369 women with primary operable breast cancer were recruited to the study between February 1, 2013 and June 6, 2018. Of these, 60 patients received treatment that was not obviously primary surgery or PET or had inadequate recorded data to make an assessment on their treatment and were excluded from the analysis. A further 455 were excluded from the analysis due to having ER negative tumours or having insufficient data to draw conclusions about their ER status. The final population for analysis included 2854 patients with ER + tumours, of whom 2354 were treated with Surgery and 500 treated with PET. See Fig. 1 for study flow diagram.

The median age of surgical patients in the study was 76 years (range 70–95) and 84 years (70–102) for the PET patients. Baseline patient and tumour characteristics are shown in Supplemental Table ST3.

### Associations with treatment type

Table 4 shows the results of the multivariable logistic regression used in the adjustment model.

**Table 4:** Multivariate logistic regression results (N = 2854).

	OR of having surgery	95% Confidence Intervals	P value
Age (per year above 70)	0.866	0.847–0.886	<0.001
ECOG PS 1∗ compared to PS 0	0.556	0.411–0.751	<0.001
ECOG PS 2∗ compared to PS 0	0.340	0.202–0.572	<0.001
ECOG PS 3∗ compared to PS 0	0.338	0.153–0.745	0.007
ECOG PS 4∗ compared to PS 0	0.294	0.023–3.699	0.343
IADL (per increase in score)	1.236	1.086–1.405	0.001
CCI (per increase in score)	0.824	0.758–0.892	<0.001
ADL (per increase in score)	1.087	0.992–1.191	0.072
Size (per mm)	0.988	0.979–0.997	0.007
Grade 2 (compared to Grade 1)	1.453	1.057–1.998	0.022
Grade 3 (compared to Grade 1)	2.607	1.665–4.081	<0.001

∗ECOG Performance Status 0: Fully active; ECOG Performance Status 1: Restricted in physically strenuous activities; ECOG Performance Status 2: Ambulatory and capable of all self-care; ECOG Performance Status 3: Capable of only limited self-care; ECOG Performance Status 4: Completely Disabled; OR = Odds Ratio; CI = Confidence Interval

Poorer performance status as assessed by the ECOG PS tool was associated with lower rates of surgical treatment. Higher rates of co-morbidity (as assessed by the CCI) and functional status (as assessed by ADL and IADL) were associated with lower rates of surgical treatment. Larger tumour size was associated with lower surgery rates, whereas patients with grade 2 or 3 tumours were more likely to undergo surgery compared to those with grade 1 disease.

### Rates of surgical treatment

**Fig. 2:**
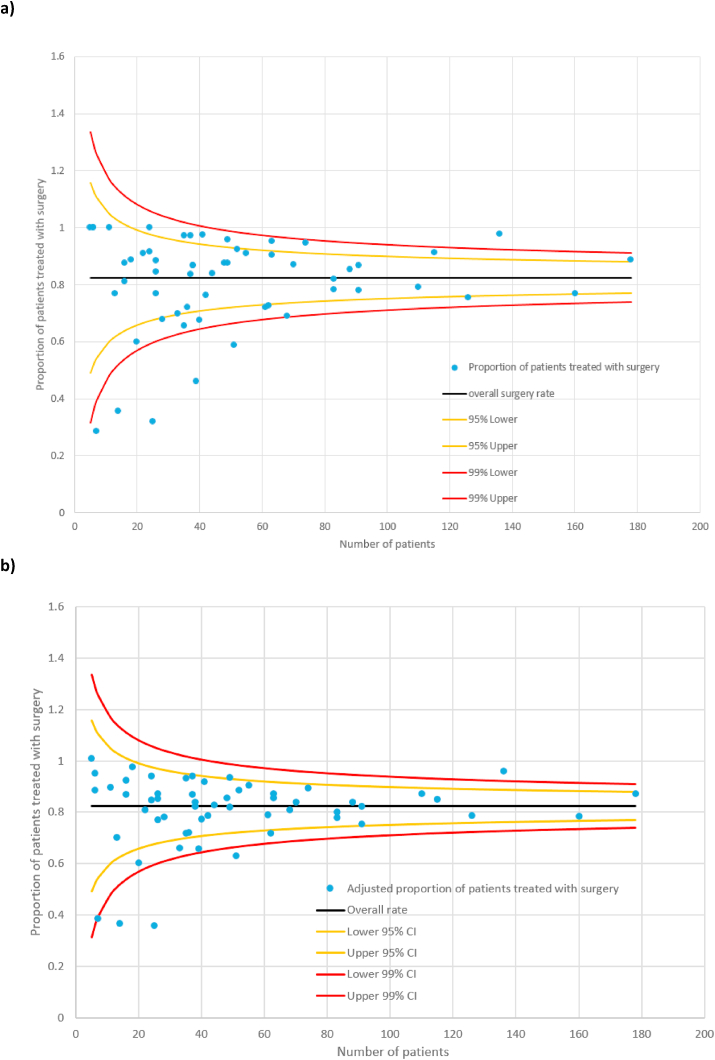
a) Unadjusted and b) adjusted rates of surgery across 56 UK breast units.

The unadjusted rates of surgery varied substantially between the 56 hospitals (Fig. 2(a)) ranging from 28.6% to 100%, with 6 of 56 (10.7%) falling outside the outer 99% limits and 23 of 56 (41.1%) falling outside the inner 95% confidence limits on the funnel plots. The expected number falling outside this limit is by defined 1% and 5% respectively. Of the 23 outlying units, 10 had a higher than expected rate of surgery and 13 had a lower than expected rate of surgery (i.e. a higher rate of PET).

Taking account of patient level characteristics and adjusting for case mix (including patient age, ECOG performance status, Charlson Comorbidity Index, Activities of Daily Living, Instrumental Activities of Daily Living, tumour size, tumour grade) reduced, but did not eliminate, the variation in surgery rates between hospitals, with 5 of 56 (8.9%) still falling outside the 99% confidence limits and 10 of 56 (17.9%) falling outside the 95% limits on the funnel plot (Fig. 2(b)). Of the 10 persistently outlying units at the 95% level, 2 had a higher than expected rate of surgery and 8 had a lower than expected rate of surgery (i.e. a higher rate of PET).

### Analysis of decision making styles

Of the study population, 2485/2854 (87.1%) patients had data available to analyse on decision-making preference, 2097 of these (84.4%) underwent surgery and 388 (15.6%) were treated with PET.

**Fig. 3:**
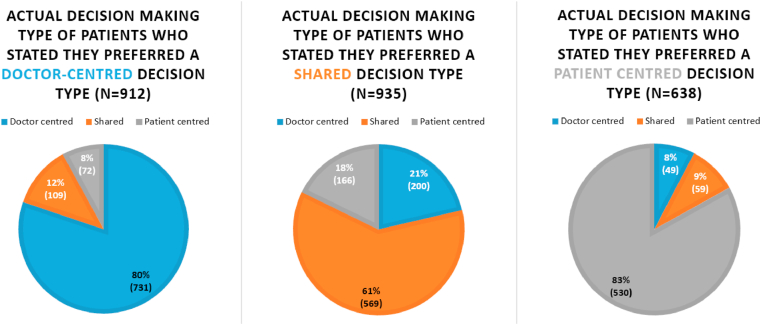
Concordance between patients' preferred and actual decision-making styles.

Patients preferred a doctor-centred decision-making style in 912 (36.7%), a shared decision-making style in 935 (37.6%) and a patient-centred decision-making style in 638 (25.7%). Patients rated their actual decision-making style as doctor-centred in 980 (39.4%), shared in 737 (29.7%) and patient-centred in 768 (30.9%). Agreement between preferred and actual decision-making style was 73.6% (Kappa = 0.60, p < 0.001; see Fig. 3).

Both preferred and actual decision-making styles were associated with final treatment type (see Table 5).

**Table 5:** Patients’ preferred and actual decision type according to treatment received.

Preferred decision type	Surgery (n = 2097)	PET (n = 388)	p	Actual decision type	Surgery (n = 2097)	PET (n = 388)	P
Doctor-centred	800	112	<0.001	Doctor-centred	872	108	<0.001
	38.1%	28.9%			41.6%	27.8%	
Shared	802	133		Shared	621	116	
	38.2%	34.3%			29.6%	29.9%	
Patient-centred	495	143		Patient-centred	604	164	
	23.6%	36.9%			28.8%	42.3%	

Patients treated with PET had significantly more patient-centred treatment decisions (42.3%) compared to shared (29.9%) or doctor-centred (27.8%); p < 0.001. Whereas patients who underwent surgery were more likely to have a doctor-centred (41.6%) treatment decision as opposed to shared (29.6%) or patient-centred (28.8%); p < 0.001 (Table 5).

Older patients had a significantly higher preference for patient-centred decision making than younger patients and this was also reflected in the actual decision type, with the youngest cohort having much more doctor-centred treatment decisions (see Table 6).

**Table 6:** Patients preferred and actual decision type by age category.

Age	70-74 (n = 940)	75-79 (n = 734)	80-84 (n = 479)	85+ (n = 332)	p-value
Preferred decision type					
Doctor-centred	358 (38.1%)	299 (40.7%)	163 (34.0%)	92 (27.7%)	<0.001
Shared	383 (40.7%)	267 (36.4%)	163 (34.0%)	122 (36.7%)	
Patient-centred	199 (21.2%)	168 (22.9%)	153 (31.9%)	118 (35.5%)	
Actual decision type					
Doctor-centred	418 (44.5%)	324 (44.1%)	154 (32.2%)	84 (25.3%)	<0.001
Shared	287 (30.5%)	194 (26.4%)	144 (30.1%)	112 (33.7%)	
Patient-centred	235 (25.0%)	216 (29.4%)	181 (37.8%)	136 (41.0%)	

Units that had a high rate of surgery (i.e. a rate higher than the upper 95% confidence limit), after adjustment for case mix, had significantly higher rates of doctor-centred actual decision-making styles (64.5% vs 30.6%; p < 0.001). Conversely, units with a low rate of surgery (i.e. a rate less than the lower 95% confidence limit) following adjustment for case mix, had significantly higher rates of patient-centred actual decision-making style (35.2% vs 11.8%; p < 0.001) (see Table 7).

**Table 7:** Patients actual decision-making style by hospital surgery rate.

	Low Surgery Rate (n = 216)	Average Surgery Rate (n = 2100)	High Surgery Rate (n = 169)	p-value
Doctor-centred	66 (30.6%)	805 (38.3%)	109 (64.5%)	<0.001
Shared	74 (34.3%)	623 (29.7%)	40 (23.7%)	
Patient-centred	76 (35.2%)	672 (32.0%)	20 (11.8%)	

## Discussion

In this large prospective cohort study of the treatment of older women breast cancer across 56 units in England, 17.5% (500/2854) of ER + patients were treated with PET, which is lower than figures published by similar recent audits; most recently, the National Audit of Breast Cancer in Older Patients found that 24% of women aged 70+ years with early ER + breast cancer were treated with PET between 2014 and 2017 [12]. This may be because our study missed recruitment of some of the older, frailer cohort that would be more like to be treated in this way due to the requirement for consented enrolment. Comparison of the study data with UK national registry data age distribution in older cancer patients does show that this study slightly over recruited younger women (70–75) and under recruited older women, so the results may not be wholly representative of the picture across the UK [34].

The analysis demonstrates that increasing age at diagnosis is associated with a reduced likelihood of receiving surgical treatment which is consistent with other similar studies [1,15,[35], [36], [37], [38]]. Higher levels of comorbidity and functional impairment (using ADL, IADL and ECOG performance status) were also associated with non-surgical treatment, which is again consistent with other published studies, where co-morbidity is often stated as a major reason for choosing PET over surgery [14,39,40]. Tumour factors were also associated with treatment type, with larger tumours being less likely to be treated surgically which may represent patients and clinicians trying to avoid more major surgery, such as mastectomy and axillary node clearance. These results corroborate and update those found by our group in a registry study of 17 129 women aged 70 years and over between 2002 and 2010 [13].

There was considerable variation in the rates of surgical treatment across the 56 hospitals and, whilst this improved with case-mix adjustment, there was still considerable variation, with 17.9% of units remaining outside the 95% limits in funnel plot analysis. Two hospitals had significantly higher and eight hospitals had significantly lower rates of surgery than could be explained by the case mix information available.

This persistence of variation in the treatment of older women with operable, ER + breast cancer at hospital level is due to factors not included in the case-mix adjustment. One possible cause is clinician or patient preference for either treatment. These results clearly show that in units with higher rates of surgery, there was a significantly higher proportion of doctor-led decision-making styles and conversely, in units with higher rates of PET there were significantly more patient-led decision-making styles.

Treatment received was strongly correlated with decision-making style, with patients choosing PET having a higher rate of patient-centred decision-making styles compared to those treated surgically. This suggests that a significant proportion of women are choosing PET as a means of avoiding surgery. It also implies that those units with high surgery rates may be more strongly promoting surgery and not taking due consideration of the preferences of women themselves. What is interesting is that a UK survey found that most breast healthcare professionals had a strong view that, if given the choice between surgery and PET, most patients would favour surgery [41], which is not what our results would suggest. Indeed, patient preference for or refusal of surgery is also often stated a reason for treatment with PET [42], however Lavelle and colleagues found, in their cohort of 800 women over the age of 70, that lower rates of surgery among elderly patients are unlikely to be due to patient choice [15]. Instead the observed variation may reflect clinician preference and how or whether alternative treatment options, such as PET, are presented at all, as was proposed by Hamaker and colleagues [43]. Current guidelines on the use of PET in the older breast cancer population state it should only be used in patients with a short life expectancy (less than 2–3 years), or when significant comorbidities preclude surgery, or in patients who refuse surgery [44,45]. It is left to the treating clinicians’ judgement as to which patients should be offered PET as an alternative treatment option to surgery. Comprehensive geriatric assessment may have a role here to help clinicians identify the patients more to benefit from being offered a choice and to support communication with patients [46].

Qualitative research in this older group of patients has suggested that they are more passive decision-makers, relying on the advice of healthcare professionals [20,21,47]. However these results clearly show there are a significant proportion of older women who prefer a shared or patient-centred decision-making style, in particular in the oldest groups. Previous studies have examined the concordance between healthcare professional and patient preference for decision-making in breast cancer patients and found that their perceptions were often inconsistent with patient preference [19]. Within this study, around three quarters of patients achieved their decision-making style, although this means a quarter did not, raising the possibility that they may be making choices which are not concordant with their treatment preferences. One of the factors which patients may prioritise highly in this age group is quality-of-life [48] and the maintenance of independence [20], both of which may be more highly preserved with PET than surgery [9], but valued less by clinicians who may innately prioritise survival metrics. In younger women the values of patients and clinicians are likely to be concordant but less so in older women. This may account for some of the discordancy observed in this study. There is also a possibility that some clinicians taking part may have adjusted their approach to information-giving in the study due to an awareness that information on decision-making was being collected.

Increasing age was associated with more patient-centred decision-making styles, both preferred and actual, which may partially explain the higher rates of PET in the oldest old. This may however be confounded by surgeons feeling more inclined to stress the importance of surgery in the youngest cohort, resulting in a perceived doctor-centred decision by patients in the younger group. Clinicians may also be aware that in the oldest and frailest women, treatment is unlikely to have a huge impact on survival as the majority of these women will die of non-breast cancer causes, so choice has less impact on survival.

This study has allowed us to collect large amounts of complex patient, tumour and treatment data on women with operable breast cancer from across the UK. Additionally, there were some issues with data completeness, with cognition and HER2 status subject to the most missing data. The well-established practice of imputation of data has therefore been used where it was deemed appropriate [49].

This study has identified outlying practice. This is important because patients who are treated with PET have been shown to have poorer outcomes compared to those treated with surgery [22,[50], [51], [52], [53]] but also overtreatment of frail older women who are unlikely to die of breast cancer regardless of treatment type may suffer unnecessary harms. Continuation of this variability in practice may result in a post-code lottery and further guidelines on the management of older women with operable breast cancer are needed. Having said that, these results do support the reports that some of the use of PET in the older breast cancer population may be due to patient choice which must be respected provided appropriate information is provided to patients to make an informed choice. There is evidence to suggest that older patients may prioritise quality-of-life over quantity [47,54], and clinicians should take this into account when counselling patients about treatment options. Shared decision-making suggests that patients should be informed of their treatment options [17] and for some older women it may be appropriate to offer PET as an alternative to ‘standard’ surgical treatment and allow the patient to decide what is best for them. Previous work by our group supports an individualised approach to treatment decision-making in this group [55] and consequently, we have recently developed, validated and trialled a decision aid to support decision making for older women facing the choice of surgery or PET [[56], [57], [58], [59]]. This tool is available on line at https://agegap.shef.ac.uk/. The tool displays health, age and fitness stratified survival outcomes for women over age 70 with early breast cancer according to whether they have surgery or PET. We hope to adapt this in the near future by adding stratified quality-of life and functional outcomes from treatment to ensure women get the information they need and value when making this choice. This may enable better evidence-based individualised decision-making and reduce variation.

## Funding

This paper presents independent research funded by the 10.13039/501100000272National Institute for Health Research (NIHR) under its Programme Grants for Applied Research Programme (Grant Reference Number RP-PG-1209-10071). The views expressed are those of the authors and not necessarily those of the NIHR or the Department of Health and Social Care.

**Previous communication to a society or meeting:** Some data were submitted and accepted for presentation to European Breast Cancer Conference March 2020 (Barcelona) and Association of Breast Surgery Conference June 2020 (Bournemouth), although both conferences were subsequently cancelled due to the COVID-19 pandemic.

## Disclosures

The authors declare no conflict of interest. Professor Stephen Walters is a National Institute for Health Research (NIHR) Senior Investigator and Jenna Morgan is a NIHR Clinical Lecturer.
