# Supplementary material for: Observational cohort study to determine the degree and causes of variation in the rate of surgery or primary endocrine therapy in older women with operable breast cancer
Source: Eur J Surg Oncol. 2021 Feb;47(2):261–8. doi: 10.1016/j.ejso.2020.09.029 (PMC7526638; doi:10.1016/j.ejso.2020.09.029)
Supplement: Multimedia component 1 [file mmc1.docx]

**Supplemental Table ST1. List of recruiting sites**

|  | **Site Name** | **Local PI** |
| --- | --- | --- |
| 1 | Sheffield | Lynda Wyld and Matt Winter |
| 2 | Barnsley | Julia Dicks |
| 3 | Doncaster | Clare Rogers |
| 4 | Milton Keynes | Amanda Taylor |
| 5 | Scunthorpe and Grimsby | Rajesh Vijh (Scunthorpe), Jenny Smith (Grimsby) |
| 6 | Leicester | Monika Kaushik |
| 7 | Derby | Kwok Leung Cheung |
| 8 | East Lancashire | Julie Iddon |
| 9 | Harrogate | Matthew Adelekan |
| 10 | St Helens and Knowsley | Riccardo Audisio |
| 11 | York | Rana Nasr (York and Scarborough) |
| 12 | Liverpool | Chris Holcombe |
| 13 | Airedale | Claire Murphy |
| 14 | Leeds | Kieran Horgan |
| 15 | Bradford | Rick Linforth |
| 16 | Cardiff | Helen Sweetland |
| 17 | Aneurin Bevan Health Board | Simon Waters (Royal Gwent), Theresa Howe (Nevill Hall) |
| 18 | Royal Lancaster | Rishi Parmeshwar |
| 19 | Coventry | Abigail Tomlins |
| 20 | Grantham | Anzors Gvaramadze |
| 21 | Lincoln | Anzors Gvaramadze |
| 22 | Pilgrim | Anzors Gvaramadze |
| 23 | Hull | Peter Kneeshaw |
| 24 | Nottingham | Lisa Whisker |
| 25 | Southport | Anwar Haq |
| 26 | Leighton | Vanessa Pope |
| 27 | Royal Marsden | Jenny Rusby |
| 28 | Cheltenham General | Sarah Vestey |
| 29 | Guys and St Thomas | Michael Douek |
| 30 | Dorset County | Caroline Osborne |
| 31 | Mid Essex | Sascha Miles-Dua |
| 32 | Mid Yorkshire | Jay Naik |
| 33 | Bristol | Zoe Winters |
| 34 | Chesterfield | Iman Azmy |
| 35 | Rotherham | Inder Kumar |
| 36 | Darent Valley | Seema Seetharam |
| 37 | Kingston | Karyn Shenton |
| 38 | Colchester | Mukesh Mukesh |
| 39 | Yeovil | Caroline Osborne |
| 40 | Croydon | Sanjay Joshi |
| 41 | North Tees | Colm Hennessy |
| 42 | South Tees | Imtiaz Cheema |
| 43 | Luton and Dunstable | Mei-Lin Ah-See |
| 44 | Weston General | Rachel Ainsworth |
| 45 | Tameside | Stephanie Ridgway |
| 46 | Macclesfield | Lisa Barraclough |
| 47 | Wrightington, Wigan and Leigh | Angela Power |
| 48 | Birmingham | Fiona Hoar |
| 49 | Kings Mill | Rebecca Boulton |
| 50 | Wythenshawe | Nigel Bundred |
| 51 | Aintree | Peter Robson |
| 52 | Brighton | Gargi Patel |
| 53 | St Margaret’s | Ashraf Patel |
| 54 | St Marys | Steve Parker |
| 55 | Oxford | Asha Adwani |
| 56 | Frimley and Wexham | Ruth Davis (Wexham), Raouf Daoud (Frimley) |
