# Supplementary material for: Observational cohort study to determine the degree and causes of variation in the rate of surgery or primary endocrine therapy in older women with operable breast cancer
Source: Eur J Surg Oncol. 2021 Feb;47(2):261–8. doi: 10.1016/j.ejso.2020.09.029 (PMC7526638; doi:10.1016/j.ejso.2020.09.029)
Supplement: Multimedia component 2 [file mmc2.docx]

**Supplemental Table ST2: Classification of decision-making styles.**

|  | **Patient’s referred decision-making style** | **Actual decision-making style** | **Decision-making Classification** |
| --- | --- | --- | --- |
| **1** | I prefer to leave all decisions regarding my treatment to my doctor | My doctor made all the decisions regarding my treatment | Doctor-centred decision-making |
| **2** | I prefer that my doctor makes the final decision about which treatment will be used, but seriously considers my opinion | My doctor made the final decision about which treatment was used, but seriously considered my opinion |  |
| **3** | I prefer that my doctor and I share responsibility for deciding which treatment is best for me | My doctor and I shared the responsibility for deciding which treatment was best for me | Shared decision-making |
| **4** | I prefer to make the final selection of my treatment after seriously considering my doctor’s opinion | I made the final selection of my treatment after I had seriously considered my doctor’s opinion | Patient-centred decision-making |
| **5** | I prefer to make the final selection about which treatment I will have | I made the final selection about which treatment I had |  |
