# Supplementary material for: Observational cohort study to determine the degree and causes of variation in the rate of surgery or primary endocrine therapy in older women with operable breast cancer
Source: Eur J Surg Oncol. 2021 Feb;47(2):261–8. doi: 10.1016/j.ejso.2020.09.029 (PMC7526638; doi:10.1016/j.ejso.2020.09.029)
Supplement: Multimedia component 3 [file mmc3.docx]

**Supplemental Table ST3: Patient and tumour Characteristics According to Treatment Type**

|  | **Treatment Type** | | |
| --- | --- | --- | --- |
|  | **Surgery**  (n=2354) | **PET**  (n=500) | **Total**  (n=2854) |
| **Age** | | | |
| **70-74 years** | **992** (42.1%) | **52** (10.4%) | **1044** (36.6%) |
| **75-79 years** | **756** (32.1%) | **79** (15.8%) | **835** (29.3%) |
| **80-84 years** | **424** (18.0%) | **142** (28.4%) | **566** (19.8%) |
| **85+ years** | **182** (7.7%) | **227** (45.4%) | **409** (14.3%) |
| **Missing** | **0** | **0** | **0** |
| **Cognition** | | | |
| **Normal** | **1399** (59.4%) | **181** (36.2%) | **1580** (55.4%) |
| **Impaired*** | **232** (9.9%) | **73** (14.6%) | **305** (10.7%) |
| **Missing** | **723** (30.7%) | **246** (49.2%) | **969** (34.0%) |
| **Charlson Comorbidity Index (CCI)** | | | |
| **3** | **891** (37.9%) | **41** (8.2%) | **932** (32.7%) |
| **4** | **529** (22.5%) | **87** (17.4%) | **616** (21.6%) |
| **5** | **457** (19.4%) | **100** (20.0%) | **557** (19.5%) |
| **6+** | **396** (16.8%) | **231** (46.2%) | **627** (22.0%) |
| **Missing** | **81** (3.4%) | **41** (8.2%) | **122** (4.3%) |
| **ECOG Performance Status** | | | |
| **Fully Active (0)** | **1659** (70.5%) | **137** (27.4%) | **1796** (62.9%) |
| **Restricted in strenuous activity (1)** | **481** (20.4%) | **167** (33.4%) | **648** (22.7%) |
| **Fully ambulatory, capable of self-care (2)** | **68** (2.9%) | **67** (13.4%) | **135** (4.7%) |
| **Capable of only limited self-care (3)** | **31** (1.3%) | **75** (15.0%) | **106** (3.7%) |
| **Completely disabled (4)** | **1** (0.04%) | **7** (1.4%) | **8** (0.3%) |
| **Missing** | **114** (4.8%) | **47** (9.4%) | **161** (5.6%) |
| **Tumour size** | | | |
| **0-10mm** | **538** (22.9%) | **50** (10.0%) | **588** (20.6%) |
| **11-20mm** | **971** (41.2%) | **178** (35.6%) | **1149** (40.3%) |
| **21-30mm** | **527** (22.4%) | **146** (29.2%) | **673** (23.6%) |
| **>30mm** | **282** (12.0%) | **114** (22.8%) | **396** (13.9%) |
| **Missing** | **36** (1.5%) | **12** (2.4%) | **48** (1.7%) |
| **HER2 status** | | | |
| **Negative** | **1640** (69.7%) | **312** (62.4%) | **1952** (68.4%) |
| **Positive** | **202** (8.6%) | **34** (6.8%) | **236** (8.3%) |
| **Inconclusive** | **70** (3.0%) | **14** (2.8%) | **84** (2.9%) |
| **Missing** | **442** (18.8%) | **140** (28.0%) | **582** (20.4%) |
| **Tumour Grade** | | | |
| **I** | **399** (16.9%) | **98** (19.6%) | **497** (17.4%) |
| **II** | **1475** (62.7%) | **329** (65.8%) | **1804** (63.2%) |
| **III** | **369** (15.7%) | **57** (11.4%) | **426** (14.9%) |
| **Missing** | **111** (4.7%) | **16** (3.2%) | **127** (4.4%) |
| **Pre-operative Lymph Node Status** | | | |
| **Negative** | **2022** (85.9%) | **406** (81.2%) | **2428** (85.1%) |
| **Positive** | **325** (13.8%) | **90** (18.0%) | **415** (14.5%) |
| **Missing** | **7** (0.3%) | **4** (0.8%) | **11** (0.4%) |

*Impaired cognition = Mini Mental State Examination score <27, known dementia or consultee participant. CCI presented here includes age component.
